# AS1411-functionalized delivery nanosystems for targeted cancer therapy

**DOI:** 10.37349/emed.2021.00039

**Published:** 2021-04-30

**Authors:** Pooria Safarzadeh Kozani, Pouya Safarzadeh Kozani, Mohammad Tariq Malik

**Affiliations:** 1 Carlos Department of Medical Biotechnology, Faculty of Medical Sciences, Tarbiat Modares University, Tehran 14115/111, Iran; 2 Department of Medical Biotechnology, Faculty of Paramedicine, Guilan University of Medical Sciences, Rasht 41446/66949, Iran; 3 Student Research Committee, Medical Biotechnology Research Center, School of Nursing, Midwifery, and Paramedicine, Guilan University of Medical Sciences, Rasht 41446/66949, Iran; 4 Departments of Microbiology and Immunology, Regenerative Medicine, and Stem Cell Biology, University of Louisville, Louisville, KY 40202, USA; 5 James Graham Brown Cancer Center, University of Louisville, Louisville, KY 40202, USA

**Keywords:** Nucleolin, AS1411, aptamer, cancer, nanoparticles, drug delivery

## Abstract

Nucleolin (NCL) is a multifunctional nucleolar phosphoprotein harboring critical roles in cells such as cell proliferation, survival, and growth. The dysregulation and overexpression of NCL are related to various pathologic and oncological indications. These characteristics of NCL make it an ideal target for the treatment of various cancers. AS1411 is a synthetic quadruplex-forming nuclease-resistant DNA oligonucleotide aptamer which shows a considerably high affinity for NCL, therefore, being capable of inducing growth inhibition in a variety of tumor cells. The high affinity and specificity of AS1411 towards NCL make it a suitable targeting tool, which can be used for the functionalization of therapeutic payloaddelivery nanosystems to selectively target tumor cells. This review explores the advances in NCL-targeting cancer therapy through AS1411-functionalized delivery nanosystems for the selective delivery of a broad spectrum of therapeutic agents.

## Introduction

Human nucleolin (NCL) is a 707-amino acid multifaceted nucleolar phosphoprotein with an approximate molecular weight of 76 kDa. It is highly expressed in exponentially growing eukaryotic cells in a proliferation-dependent manner. Its presence in the dense fibrillar core and granular regions of the nucleolus is regarded to be higher than any other protein [1, 2]. NCL harbors a tripartite structure comprised of an acidic NH2 terminus involved in numerous protein-protein interactions, a central globular domain with four RNA-binding domains involved in pre-RNA processing, a COOH terminus domain comprising arginine-glycine-glycine repeats which interacts with ribosomal proteins, and four phosphorylation sites [3]. The primary function of NCL in the nucleus is the regulation of rRNA synthesis and ribosome biogenesis. It acts as a shuttling protein between the cytoplasm and the nucleus and promotes the import of ribosomal subunits to the nucleus [2].

NCL dysregulation has been observed in numerous pathological conditions such as autoimmune disorders, Alzheimer and Parkinson’s disease, and numerous cancer types [4–8]. It has been evident that NCL is capable of inducing the maturation of some cancer-related miRNAs [9], acting as an adhesion molecule modulating cell-matrix interaction in angiogenesis, regulating cell migration [10], functioning as an anti-apoptotic element by acting as a BCL2-stabilizing factor [11–13], and acting as a macrophage receptor for early apoptotic cells [14].

NCL plays substantially imperative tasks in the modulation of cell proliferation, survival, and growth, cytokine production, and nuclear biogenesis [1, 15]. Furthermore, it holds various other multifaceted responsibilities in the cellular occurrences such as DNA recombination, as well as transcription, packing, and transportation of rRNA [1]. Such molecular tasks include roles in the biosynthesis of ribosomes, RNA binding, and DNA and RNA helicase activity [15]. NCL also interacts with manifold mRNAs, promoter regions of c-Myc [8, 16, 17], and microRNA biogenesis microprocessor machinery [9, 18]. The function of NCL is regulated by diverse molecular mechanisms at different stages, such as phosphorylation [1, 19–21], ADP-ribosylation, methylation [15], and SUMOylation [22]. Alongside being famous as a nucleus-related protein, NCL is also capable of operating as a shuttle molecule as well as acting as a membrane-anchored receptor [23]. The physiological functions of NCL include chromatin remodeling and the maintenance of embryonic stem cells by blocking the expression of p53. NCL also supports both microtubule organization at centrosomes and malignant transformation. At the cell surface, NCL activates extracellular signal-regulated kinases upon interacting with C-X-C motif chemokine receptor 4 (CXCR4) [24].

Aptamers are approximately 20- to 60-nucleotide deoxyribonucleic acid, ribonucleic acid, or xeno nucleic acid constructs capable of binding craved molecules with robust a capability and specificity by folding into tertiary structures [25]. They are non-immunogenic and innocuous nucleotide equivalents of antibodies [26], which makes them irreplaceable candidates for various fields of applications from clinical diagnostic and therapeutic applications to the purification of desired molecules [25, 27, 28]. Because of their diverse superiorities, aptamers have various applications in different fields such as in biosensors, therapeutics, and diagnostics [25].

Systematic Evolution of Ligands by EXponential Enrichment (SELEX) is a standard method of the generation of new novel aptamers for any particular target of interest. In this approach, a pool of 10^14^ to 10^16^ single-stranded random 40 to 100 long oligonucleotides are incubated with the target of interest. These oligonucleotides exhibit different binding affinities to the target of interest. Therefore, throughout the process, the oligonucleotides that have a low binding affinity to the target are excluded and, in the end, only a number of oligonucleotides remain which have a high level of binding affinity for the target of interest. Recently, various SELEX-based methods have been proposed and utilized for the development of aptamers for novel targets with considerable specificity and sensitivity. These techniques include capture-SELEX, capillary electrophoresis-SELEX (CE-SELEX), cell-SELEX, atomic force microscopy-SELEX (AFM-SELEX), artificially expanded genetic information system-SELEX (AEGIS-SELEX), and immunoprecipitation-coupled SELEX (IP-SELEX) [29].

The structural diversity of ribonucleic acid aptamers is noticeably greater than deoxyribonucleic acid aptamers, however, their applicability is hindered by their degradability by distinctive elements including RNases or heat [30, 31]. Setting aside the similarities of aptamers with antibodies, they also possess numerous superiorities over antibodies including being smaller, immune-compatible, and less time-consuming to produce, and having higher tissue-penetration, manufacturing affordability, and pronounced thermal stability [25, 32]. Among all the mentioned advantages, aptamers also suffer from some hindrances limiting their broader application, of which their non-specificity, interaction with intracellular molecules, and elimination from the bloodstream as well as rapid degradation can be mentioned [32]. The extraordinary specificity and sensitivity level, being non-immunogenic, the easy and cost-effective production process, the high range of potential targets with high affinity from a variety of molecules, and the easy and simple process of applying chemical modifications to them for making them resistant to enzymatic reactions are all among worthwhile advantages of aptamers in comparison with other types of targeting tools [33]. Considering these pros, unfortunately, aptamers tend to have cons as well, such as their susceptibility to enzymatic reactions that endangers their ability for nanoparticle (NP) redirection, producing negative charges on the surface they have been positioned on, and their time-to-time off-target caveats. However, studies have proposed that many of these drawbacks can be solved by using chemical modifications approaches or using meticulous equipping tactics for putting these targeting moieties on the surface of NPs [33]. As every other targeting moiety, aptamers also have the off-target limitation which has been in the center of attention for addressing in a number of studies. For example, some researchers have suggested adding a SELEX negative selection step in the production of aptamers. In this step molecules similar to the real target of the aptamers are used and the aptamers reactive to the similar molecules with high affinity are excluded from the process of selection and they will not be considered as the final aptamer candidate [32]. Furthermore, other researchers have stated that chemical modification can also be exploited for elevating the targeting specificity of aptamers [34, 35]. Elskens et al. [34] have indicated that pre-SELEX and post-SELEX modifications including truncation, making bivalent and multivalent aptamers, modifying aptamers with various crosslink moieties (such as phenyl azide, 5-iodo deoxyuridine, diazirine, aldehyde, and F-carboxyl), phosphodiester modifications (thioaptamers), nucleobase modifications [such as C5 modifications (SOMAmers) and expanded genetic alphabet], and sugar modifications such as 2’ fluoro arabino nucleic acid (2’F-ANA), and locked nucleic acids (LNA) can improve the targeting capacity of aptamers very efficiently. These methods have proven very efficient in addressing the off-target limitation of aptamers and they can help aptamers maintain their physiological properties.

Aptamers have been in the center of attention for the treatment of cancer and various other diseases. For instance, pegaptanib sodium (Macugen) has been approved by Food and Drug Administration (FDA) for treatment of age-related macular degeneration (AMD) [36]. Furthermore, AS1411 and NOX-A12 are two other successful aptamers that are currently under clinical investigation [36]. Moreover, other aptamers such as AX102, xPSM-A10 (A10), HB5, HeA2_3, MP7, and aptPD-L1 are other examples of successful aptamers that are under preclinical investigations for their potential application in cancer treatment modalities [36].

AS1411 (formerly named AGRO100) is a manufactured 26-bp unmodified phosphodiester oligonucleotide that is capable of forming a highly stable nuclease-resistant four-stranded dimer that inhibits the growth of transformed cells and a wide spectrum of malignant cell lines [37, 38]. AS1411 is the first anticancer aptamer investigated in Phase I and II human clinical trials [37]. AS1411 was discovered by Bates and co-workers, and it was revealed that it is competent to target NCL with accentuated capability, which has led to different studies investigating its potential growth inhibitory impacts on a variety of cell lines [37, 39].

Chemotherapy and radiotherapy are regarded as the most prevalent cancer treatment approaches but they can lead to serious side effects in cancer patients [40]. In the past decades, targeted cancer therapy has gained attention because of its specific way of promoting cytotoxicity. One of the most renowned approaches for targeted cancer therapy is based on antibodies [such as antibody-drug conjugates, bispecific T-cell engagers (BiTEs^®^) or chimeric antigen receptor (CAR) T cell therapy] [41–46]. Even though antibody-based treatment modalities are highly specific with diminished side effects, they might still face obstacles in their clinical application because of their probable immunogenicity and manufacturing expenses [41–46]. On the other hand, aptamer-based targeted therapeutics and specific drug delivery platforms have recently shown that they can overcome the mentioned challenges. Therefore, aptamer technology evidently has various superiorities over protein-based antibody therapies as it will be described in this review. The main idea behind nanosystems is to safely deliver the cargo molecules to the target site of interest with the minimum amount of side effects and off-target events. Additionally, nanosystems can be efficiently utilized for the delivery of various types of cargoes such as drugs, genes, and other therapeutic molecules of interest. It is very fascinating how fast the use of nanosystems for the delivery of various cargoes has changed the face of selective drug delivery in the past years. One other factor that has highlighted the importance of this modality is its ability and tunability for the controlled release of its cargo in the site and at the time of interest. Furthermore, cargo delivery nanosystems can also protect their cargo against unfavorable situations which might negatively affect various properties of that cargo.

**Figure 1.**
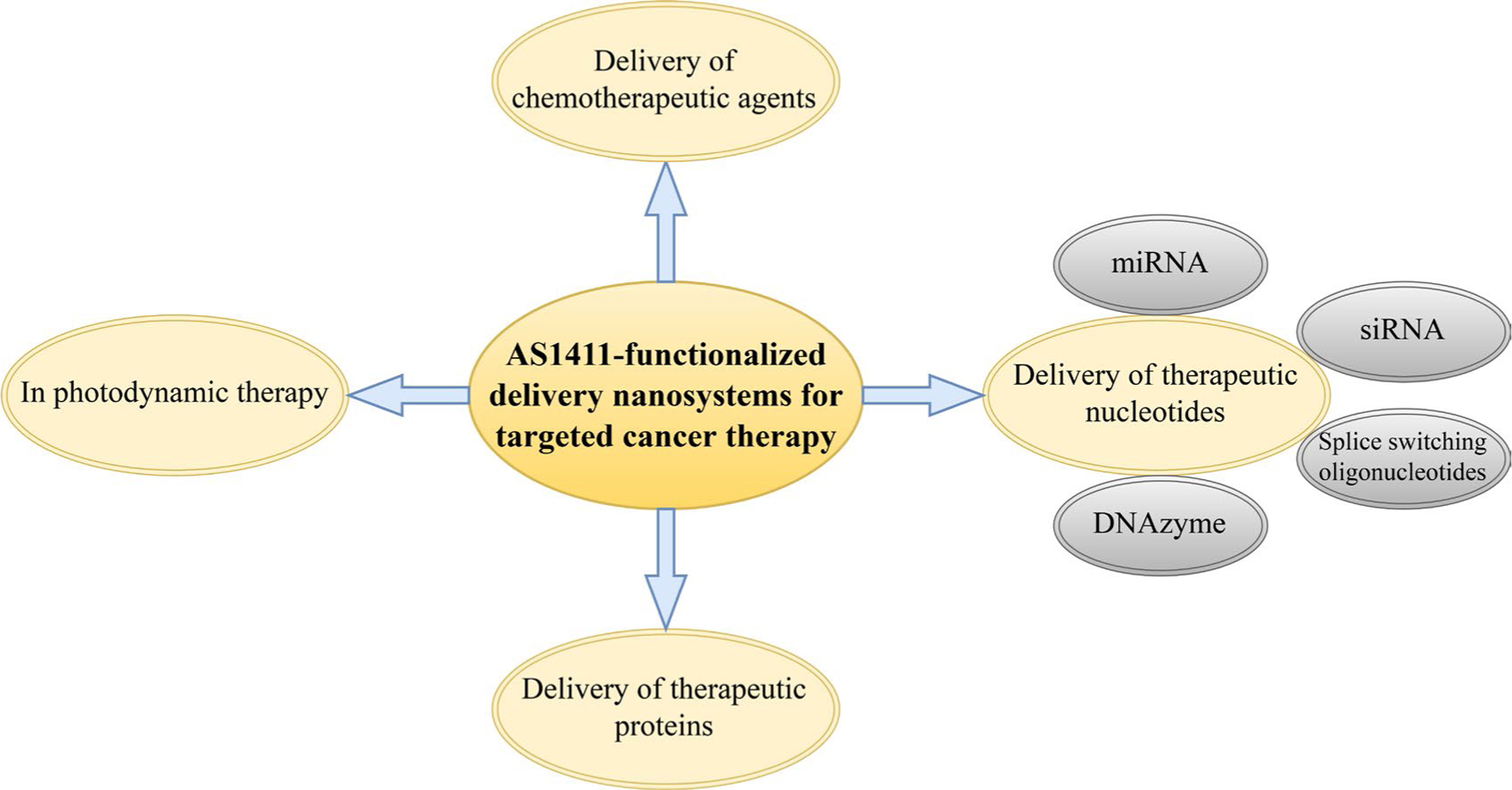
An overall representation of the topics discussed in this review

In this review, we discuss different types of AS1411 aptamer-functionalized nanosystems for the delivery of chemotherapeutic agents, therapeutic nucleotides, and therapeutic proteins to different types of malignant cells overexpressing NCL. We also briefly discuss how these nanosystems can be exploited for enhancing photodynamic therapy (PDT) (Figure 1). Ultimately, we demonstrate that NCL is a great target for novel cancer therapy approaches using AS1411 aptamer-functionalized nanosystems since it is a common feature of various types of tumor cells.

## Delivery systems

### Chemotherapeutic delivery

Combinational micelle therapeutic agent delivery platforms equipped with the anti-NCL aptamer can be applied for the precise delivery of different chemotherapeutics including doxorubicin (DOX) to various malignant cells. The elaborate system described in this section somehow manages to tackle the poor drug-loading capacity and micelle stability issues through the development of an AS1411-modified hybrid system consisting of Pluronic F127 and beta-cyclodextrin-linked poly(ethylene glycol)-b-polylactide (β-CD-PELA) block copolymers as co-carriers of the anticancer drug [47]. Pluronic F127, which is an amphiphilic polymer extensively used in nano-drug delivery systems, tends to self-assemble into micelles while it also possesses terminal hydroxyl groups which can be readily functionalized for bioconjugation purposes [48, 49]. Despite the ideal characteristics of Pluronic F127, it is still entwined with several downsides in terms of micelle formulation, such as poor physical stability and drug-loading capacity, which can be tackled while utilized alongside other copolymers (such as Pluronic P123 and β-CD-PELA) in the development of combinatorial systems [47, 50]. β-CD-PELA block copolymers are capable of forming self-assembled micelles with low critical micelle concentration and enhancing the drug-loading capacity due to the combined hydrophobicity of both polylactide (PLA) blocks and the inner cavity of β-CD [51]. Therefore, the proposed construct (composed of both Pluronic F127 and β-CD-PELA) can exhibit an improved profile of physical stability and agent-loading capacity alongside exhibiting enhanced cellular uptake (due to NCL-mediated endocytosis), prolonged circulation duration, elevated accumulation in tumor cells which consequently results in enhanced tumoricidal activity, and minimized cardiotoxicity [47]. These ideal characteristics demonstrate that aptamer-conjugated combinational micelles with versatile functions might serve as potential delivery vehicles for anticancer purposes [47].

Another investigation has described the establishment of a cancer-specific delivery system for the selective cytoplasmic release of paclitaxel (PTX) in ovarian malignant cells based on a combinatorial micellar construct. This construct is made up of (A) a pH-responsive copolymer synthesized by a condensation polymerization reaction in the presence of tocopheryl polyethylene glycol 1000 succinate (TPGS)-diacrylate macromonomer and (B) an AS1411 aptamer-decorated TPGS polymer (AS1411-TPGS). As proposed by Zhang et al. [52], the incorporation of AS1411-TPGS copolymers on micelle surfaces improves cancer cell recognition through the presence of NCL on the plasma membrane of cancer cells while the encapsulation of PTX in the AS1411-mixed micelles results in the quick release of the drug in a weakly acidic environment with a pH of 5.5 [52]. PTX/AS1411-mixed micelles exhibit significantly increased internalization only into cancer cells and not healthy ones which is because of their AS1411-NCL interaction-mediated enhanced transmembrane ability leading to a significantly increased tumor accumulation of PTX which consequently results in elevated cytotoxicity, G2/M phase arrest, and tumor growth inhibition [52]. In general, this dual-functional Apt-mixed micellar system might serve as a promising and potent targeted drug delivery system for anticancer purposes [52].

Furthermore, other researchers have proposed a novel pH-reactive micelle-based delivery system for the delivery of DOX for effective breast cancer therapy [53]. These DOX and AS1411 encapsulated pH-reactive delivery nanoparticles (PRNs), which exhibit spherical shapes and favorable colloidal characteristics, are composed of biocompatible polyethylene glycol (PEG)-poly(β-amino esters) (PAEs) NPs synthesized by Michael addition polymerization [53]. In an aqueous solution with a pH of 7.4, PEG-PAEs are automatically structured into micellar constructs in the hydrophobic core of which the hydrophobic agent is encapsulated. This leads to the formation of the drug-loaded NPs. Eventually, electrostatic interactions mediate the immobilization of the negatively charged AS1411 aptamer on the hydrophilic surface of the NPs leading to the formation of co-delivery NPs [53]. Further on, PRNs are concentrated in the tumor tissue by the enhanced permeability and retention (EPR) effect and then they are internalized into the tumor cells by AS1411-mediated endocytosis alongside the contribution of the positive charges on the surface of the PRNs [53]. After entering the intracellular endosomes or lysosomes, pH-triggered drug release is mediated by the acidic environment of the compartments, which results in micelle disintegration and subsequent drug release, thus improving the localization and cytotoxicity of DOX [53]. This type of multifunctional nanomicelle-based delivery approach offers a highly specific targeting ability, which is only towards tumor cells and not normal cells, and it might be further applied for enhanced drug delivery in cancer treatment modalities [53].

**Figure 2.**
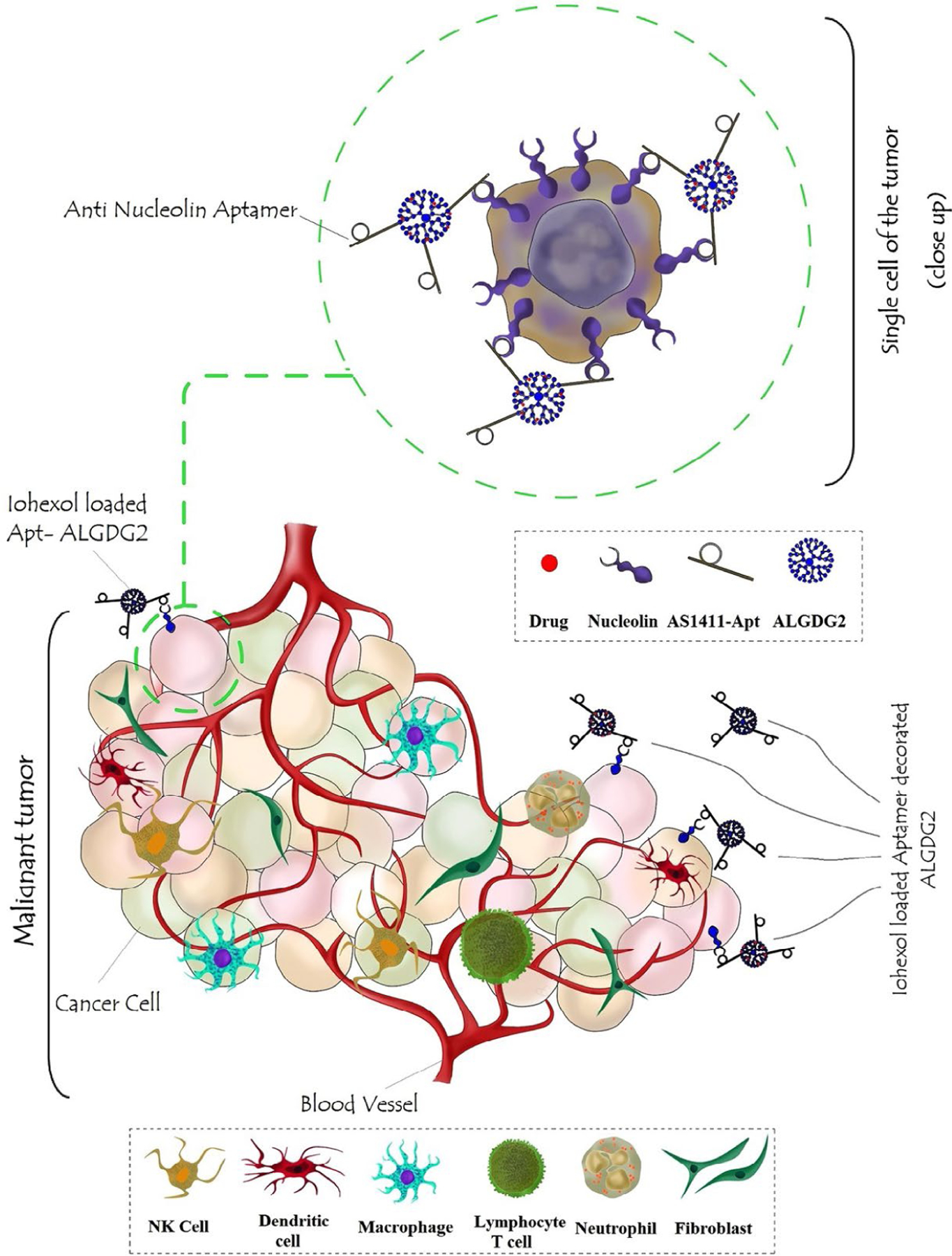
A graphic illustration of AS1411-functionalized ALGDG2 loaded with iohexol *Note.* Reprinted with permission from “AS1411 aptamer-anionic linear globular dendrimer G2-iohexol selective nano-theranostics” by Mohammadzadeh P, Cohan RA, Ghoreishi SM, Bitarafan-Rajabi A, Ardestani MS. Sci Rep. 2017;7:11832 (https://doi.org/10.1038/s41598-017-12150-8). CC BY.

Another contribution to the topic has been conducted by Mohammadzadeh et al. [54] as they developed a selective nano-theranostic system composed of AS1411-functionalized anionic linear globular dendrimer G2 (ALGDG2) for the specific delivery of Iohexol to breast cancer cells. ALGDG2 is generally considered as an ideal carrier for a broad spectrum of anticancer, antiviral, and imaging agents due to its low molecular weight, low toxicity, immunocompatibility, biodegradability (because of its sidelong citric acid groups), high purity and hydrophilicity, monodispersity, favorable permeability to cancer cells, and possession of various well-known functional groups on the spherical particles’ surface [55–58]. Taken together, these nano constructs exhibited a considerable potential for reducing the number of cancer cells by maximizing the accumulation of iohexol in the tumors while they minimized the toxicity of Iohexol on normal cells [54]. A graphical scheme of this nano-theranostic has been represented in Figure 2. Also, a summary of different AS1411-functionalized nanosystems developed for the delivery of chemotherapeutic agents has been represented in Table 1.

### Therapeutic oligonucleotide delivery

#### miRNA delivery

AS1411-aptamer-mediated targeted delivery can also be used for the delivery of an antimiR for the inhibition of miRNA-221 which has been known to be involved in the cancer development of papillary thyroid carcinoma in which it is highly expressed [72, 73]. A NCL-targeting theragnostics probe (hereafter referred to as MFAS miR-221 MB) based on AS1411-armed MF NPs equipped with miRNA-221 molecular beacon (miR-221 MB), which is complementary to miRNA-221, for simultaneous targeting of various tumor cells, intracellular imaging of miRNA-221 biogenesis, and disruption of miRNA-221-mediated carcinogenesis might be considered a successful and potent antitumor therapeutic and diagnostic platform with considerable cancer-type flexibility which is achievable through the changing of the targeted miRNA [74]. The MFAS miR-221 MB is internalized into the cytoplasm upon binding to NCL on the surface of cancer cells. After internalization, the reductive environment of the cytoplasm mediates the cleavage of the disulfide linkage between MF and miR-221 MB, which results in the consequent unloading of the miR-221 MB within the tumor cells [74]. In the presence of miRNA-221 in the cytoplasm of tumor cells, which is directly responsible for the oncogenic down-regulation of various tumor suppressor genes, miR-221 MB will hybridize with it which results in a loss of functions of miRNA-221, thus disrupting the miRNA-221-dependent carcinogenesis through the overall reduction of the expression of oncogenes [74]. Additionally, the researchers of this study have also indicated that MFAS miR-221 MB can also be utilized to image intracellularly expressed miRNA-221 [74]. In detail, they demonstrated that the miR-221 MB separation from the MFAS miR-221 MB complex in the cytoplasm of C6 glioma cells evidently images miRNA-221 biogenesis [74].

#### DNAzyme delivery

The onset of cancer causes cancer cells to overexpress a variety of proteins known as inhibitors of apoptosis (IAPs), which enable them to increase their life-span while minimizing the chance of apoptosis occurrence. Survivin (Sur), as an example of IAPs, which is highly overexpressed in the nucleus, cytoplasm, and cellular organelles such as the mitochondria of various cancer types including retinoblastoma (RB), is responsible for mediating various oncogenic signaling pathways, inhibition of apoptosis, rapid cell growth, and eventually as the name indicates the survival of tumor cells [75–78]. RB is a rare form of childhood cancer caused by mutations in the *RB* gene or both allele inactivation, which impacts the cell cycle and apoptosis regulators of immature retinal cells [79]. Because of the roles of Sur in tumor recurrence, diminished patient survival, and chemotherapy and radiotherapy resistance alongside its overexpression in RB tumor cells and its secretion and presence in the serums of the patients, it is regarded as one of the most suitable targets for the treatment of RB [80–83].

An aptamer-mediated NCL-targeting approach for the delivery of survivin DNAzyme (Sur_Dz) can act as a way of specific gene-targeting therapy to suppress the progression of RB cancer. A chimeric conjugate composed of the AS1411 aptamer (NCL-APT) and Sur_Dz with poly T linker at the 5’ end of the aptamer followed by complementary bases to Sur_Dz has been developed by Subramanian et al [84]. This construct could be an efficient delivery platform without compromising the functional activity of the Dz in cleaving Sur mRNA [84]. This novel chimeric aptamer-DNAzyme conjugate (NCL-APT-Sur_Dz) demonstrates itself as promising and powerful therapeutics for targeted combating cancer cells through the down-regulation of Sur expression and functionality and consequently allowing for the occurrence of apoptosis in the absence of its inhibitor [84].

Since both Sur and NCL are overexpressed in a variety of cancers, the chimerization of NCL-APT with Sur_Dz for the development of a targeted therapeutic platform can be functional on a wide spectrum of cancer types. Furthermore, Sur_Dz has only been chosen due to its dose-dependent manner catalytic reactivity towards Sur mRNA which has been proven in pancreatic carcinoma and its overexpression profile in RB tumor cells [84, 85]. Moreover, the flexibility of this delivery system allows for the delivery of different Dzs which can specifically perform anticancer catalytic reactions towards specific targets of interest in various other cancer types.

#### siRNA delivery

Non-small cell lung cancer (NSCLC) is one of the cancer types that causes a high mortality rate in lung cancer patients due to its early metastasis events which are composed of several consecutive stages including epithelial-mesenchymal transition (EMT), cancer cell migration, invasion, intravasation into the systemic circulation, eventual adhesion to endothelial cells, and extravasation and colonization of distant organs as well as induction of angiogenesis [86–88]. The activation of key metastatic signaling cascades and the promotion of malignant transformation in NSCLC have been known to be associated with the overexpression of snail family zinc finger 2 (SLUG) which is a zinc-finger-containing transcriptional factor that mediates the activation of EMT, migration, and invasion of lung cancer cells and neuropilin 1 (NRP1) which mediates the upregulation of the matrix metalloproteinases-2 (MMP-2) expression and activity resulting in an increase in tumor cell invasion, angiogenesis, and distant organ colonization [89–95]. Considering the known functions of SLUG and NRP1 in invasion and metastasic capabilities of NSCLC, they are deemed as suitable targets for the blocking of key oncogenic signaling pathways [89–95].

NCL aptamer-siRNA (AS1411-siRNA) chimeras specific for SLUG (aptNCL-SLUGsiR) and NRP1 (aptNCL-NRP1siR) can be used for specific tumor invasion and angiogenesis suppression in only metastatic tumor cells without blocking the signaling pathways of cells that are under physiologic conditions [96]. AptNCL-SLUGsiR- and aptNCL-NRP1siR-mediated suppression of SLUG and NRP1, respectively, can decrease cell growth, motility, invasiveness, and angiogenesis of only NCL-expressing cancer cells which demonstrates that synergistic suppression of lung cancer can be achieved using a combination of both aptamer-siRNA chimeras [96]. In general, this strategy offers a cancer-specific targeting approach with concurrent gene-specific silencing capabilities [96].

Additionally, melanoma is a common type of skin cancer that is developed from the pigment-producing cells melanocytes and is difficult to treat due to the high rate of its early-stage metastasis, poor prognosis, and resistance to conventional radiotherapy [97, 98]. In a majority of melanomas (almost 60%), melanocyte biology and disease pathology are significantly influenced by the expression of the mutant forms of the *BRAF* gene, such as *BRAFV599E*, and the activation of the RAS/RAS/MAPK pathway which happens to be essential for melanoma cell viability and transformation [99]. The important oncogenic role of the mutant *BRAF* gene expression makes it a suitable target for the treatment of melanomas through siRNA-mediated gene silencing approaches. Since liposomes are non-viral carriers employed for the development of successful drug delivery systems, some of which have already been approved by the US FDA for medical use, they can be employed for the efficient delivery of gene-modifying agents into cells [100, 101]. PEGylated cationic liposomes conjugated to AS1411 aptamer (AS1411-PEG-liposome, designated as ASLP) and equipped with anti-BRAF siRNA (siBraf) can might be utilized as a tumor-targeting gene-silencing delivery system (ASLP/siBraf) against melanomas since they can exhibit significant silencing activity against the *BRAF* gene and inhibit melanoma growth [102].

**Figure 3.**
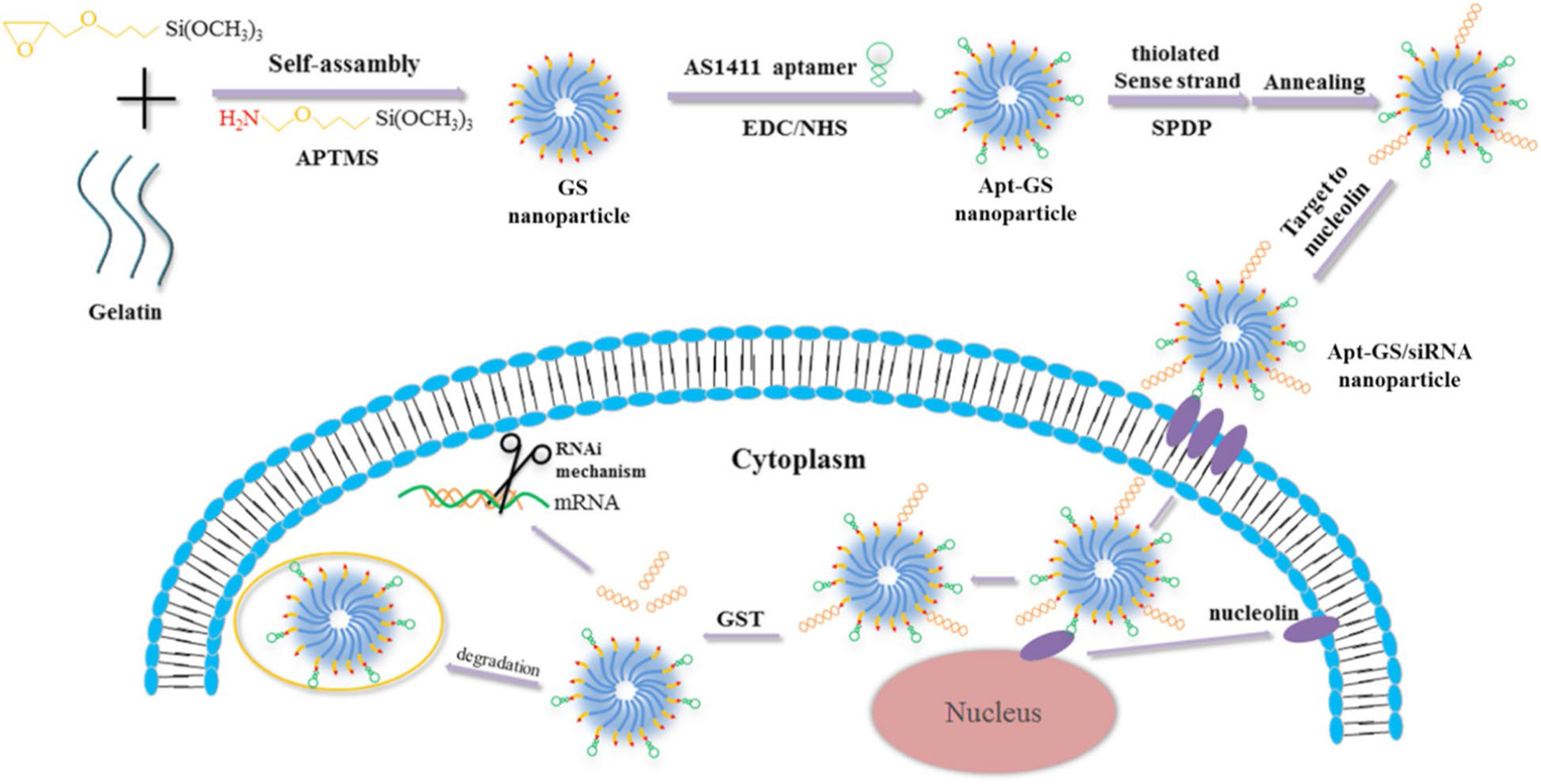
Redox-reactive gelatin/silica-based nanogels functionalized with AS1411 for the selective delivery of disulfide-conjugated siRNA. APTMS: 3-aminopropyl-trimethoxysilane; SPDP: N-succinimidyl 3-(2-pyridyldithio) propionate; EDC: ethyl(dimethylaminopropyl) carbodiimide; NHS: *N*-hydroxysuccinimide; Apt: aptamer; GST: glutathione *S*-transferase *Note.* Reprinted with permission from “Redox-sensitive gelatin/silica-aptamer nanogels for targeted siRNA delivery” by Zhao X, Xi Y, Zhang Y, Wu Q, Meng R, Zheng B, et al. Nanoscale Res Lett. 2019;14:273 (https://doi.org/10.1186/s11671-019-3101-0). CC BY.

Additionally, another study has reported the development of redox-reactive gelatin/silica-based nanogels functionalized with AS1411 for targeted siRNA delivery (Apt-GS/siRNA) which were transiently conjugated to smart nanogels via a disulfide linker [103]. Since tumor cells approximately contain ten-fold higher glutathione (GSH) concentration than normal cells, Apt-GS/siRNA nanogels exhibit significantly selective cytosolic release of functional siRNA mediated by disulfide cleavage in the presence of GSH (Figure 3) [103–105]. Taken together, this redox-reactive gelatin-based smart nanogel system exhibits considerable potential for the effective delivery and GSH-triggered release of siRNAs which can be considered for RNAi-mediated tumor elimination [103].

In addition to the mentioned studies, an AS1411 aptamer-functionalized nanoliposome-based delivery system has been developed for the co-delivery of PTX and polo-like kinase 1-targeted siRNA (PLK1-targeted siRNA) to breast cancer cells [106]. PLK1 is a highly conserved serine/threonine protein kinase with important regulatory mitotic effects whose high expression levels have been significantly associated with abnormal tumor cell proliferation, metastasis, angiogenesis, and tumor prognosis in various cancers such as breast cancer [107, 108]. Therefore, PLK1 can be considered as a promising primary target candidate for cancer treatment modality, such as PLK1-targeting RNAi-based gene therapy [106, 109–111]. The simultaneous co-delivery of PTX and siRNA proposed by Yu et al. [106] results in a synergistic incremental pattern of apoptotic cells and diminished angiogenesis. Such effects are considered advantages over the effects mediated by the separate delivery of PTX and siRNA which might demonstrate the potential of this delivery system for clinical investigation [106].

#### Splice-switching oligonucleotides

Splice-switching oligonucleotides (SSOs) are short synthetic single-stranded oligonucleotides capable of binding to a splice site or splicing enhancer of a pre-mRNA, thus preventing the endogenous splicing machinery from binding to those splice sites which can eventually result in the disruption of the normal splicing repertoire and the generation and subsequent translation of alternative versions of a mature mRNA [112–115]. SSOs are considered as potent and powerful tools for the generation of phenotypic changes in cells because of several major advantages (such as their nuclease resistance characteristics due to the possession of a phosphorothioate backbone) of this antisense-based technology over other mRNA knockdown approaches such as siRNA [112, 115–117]. One of the other therapeutic advantages of SSOs is that only a small percentage of pre-mRNAs need to be properly spliced so that a phenotype correction associated with multiple genetic mutations can come into effect. For example, apoptosis induction can happen after SSO-mediated switching of only a small percentage of Bcl-xL to Bcl-xS [118–120].

Since the ability of AS1411 for internalization into multiple cancer cells and migration to their nucleus has been evident, the therapeutic applicability of SSOs can be further exercised by their targeted delivery to the nuclei of specific cells of interest [112, 116, 117, 121, 122]. This goal can be achieved through the ability of SSOs to be easily appended to aptamers because of their 2’-*O*-methyl-phosphorothioate [112, 116, 117, 121, 122]. Here we report the engineering of NCL aptamer-based chimeras carrying cargoes of SSOs for the selective delivery of these therapeutic oligonucleotides to the nuclei of tumor cells that express NCL [123].

Modulation of nuclear events through the enhancements in splice correction of pre-mRNA can be achieved using aptamer-splice-switching oligonucleotide chimeras [123]. It has been indicated that splicing alternations effectively occur in much lower doses of aptamer-SSO chimeras than the SSO alone (a fact that further demonstrates the efficacy and safety of this antisense-based therapeutic approach) [123]. Kotula et al. [123] have engineered their aptamer-SSO chimeras in a way that they possess a double-step selectivity that further expands their safety index with the aptamer domain being able to specifically bind to an overexpressed receptor on the surface of multiple cancer cell types of interest and the therapeutic splice switch which is only capable of impinging on the essential pathways of cancer cells (but not healthy ones). Since such aptamer chimeras are capable of delivering cargos to the nucleus and nucleolus of target cells while avoiding endosomal compartments, they can be further utilized for various other nucleolar event changes (such as sequestering genes into heterochromatin, ribosome biogenesis, or even the selective delivery of pro-apoptotic SSOs to tumor cells of interest for therapeutic tumoricidal applications) [123]. In a nutshell, aptamer-SSO chimeras are capable of internalizing target cells through more than one internalization pathway, which can be dynamin-independent, and they might offer an affordable therapeutic approach while minimizing unwanted adverse events [123].

### Therapeutic protein delivery

Lactoferrin is an innocuous natural multifaceted glycoprotein, primarily identified in bovine and human milk, with various characteristics such as immunomodulatory and anticancer properties which has been regarded safe for consumption [124–127]. Nowadays, the native form of cow milk-derived lactoferrin has gained worldwide attention for therapeutic applications [124, 125, 127]. In one study, experiments have been conducted to validate the tumoricidal efficacy of the bovine lactoferrin that is saturated with iron (bovLfn^Fe^) in combination with multimodal imaging efficacy of Fe_3_O_4_ NPs which are hereafter referred to as bovLfn-Fe_3_O_4_ [128]. Chitosan-modified calcium phosphate nano-constructs functionalized with epithelial cell adhesion molecule (EpCAM)- and NCL-specific aptamers were used by Roy et al. [128] for the encapsulation of bovLfn-Fe_3_O_4_ to achieve tumor-specific uptake of the constructs. This mentioned nanoformulation (bovLfn-Fe_3_O_4_ nano-constructs) achieved considerably encouraging tumor rejection in triple-positive (EpCAM, CD133, CD44) colon cancer xenograft mouse models inducing higher survival rates in comparison with non-targeted nano-constructs which demonstrates the importance of nano-constructs functionalization with the anti-NCL and anti-EpCAM aptamers [128]. The tumor suppression mechanism of bovLfn-Fe_3_O_4_ nano-constructs occurs as the extracellular death domain receptors of TRAIL and Fas mediate the phosphorylation and subsequent activation of p53 and the Notch pathway inhibition [128]. The activation of p53 induces the activation of Bad and mitochondrial depolarization resulting in the release of SMAC/DIABLO and cytochrome C [128]. Eventually, apoptosis induction is mediated by the inhibition of the Akt pathway and the release of cytokines such as interleukin 27 (IL-27) and keratinocyte chemoattractant (KC) from monocytes/macrophages and dendritic cells [128]. Furthermore, the inhibition of pro-angiogenic markers (such as Amphiregulin, FGF, GM-CSF, and TIMP-4) mediate the process of angiogenesis suppression [128]. In a nutshell, bovLfn-Fe_3_O_4_ nano-constructs combine the anticancer potential of bovLfn with the efficacious multimodal imaging capabilities of Fe_3_O_4_ as they can completely inhibit tumor growth without exhibiting signs of adverse events alongside showing immunomodulatory benefits through increasing red blood cells, hemoglobin, and zinc levels [128].

### PDT

Ai et al. [129] have investigated the synthesis and application of AS1411-customized fluorescent Au NPs. The unique association between AS1411 and NCL on malignant cells allows the resultant nano-constructs to specifically bind to such cells, therefore, they can be used for both targeted cancer cell imaging and PDT [129]. In brief, the irradiation of the nano-constructs can result in the efficient production of cytotoxic reactive oxygen species leading to fatal cellular damages [129]. Moreover, not only the nano-constructs inherently possess particular cytotoxicity towards malignant cells, but they also accentuate the cellular uptake of the fluorescent groups which in turn leads to the maximized efficiency of both the targeted cancer cell imaging and PDT [129]. This strategy with its simplicity and affordability can also be utilized as a qualitative method for the recognition of the presence or absence of malignant cells besides having the potential to be used as a semi-quantitative method to measure their population based the fluorescence of the cell imaging [129].

Another study has proposed a similar method with minor differences. In this novel strategy, as a drug carrier to target malignant cells for PDT, several molecules of porphyrin derivative are physically conjugated to AS1411 to form the apt-TMP complex [130]. Porphyrin derivatives are broadly applied in cancer PDT. However, it has been shown that they cause side effects towards some normal cells [131]. So, systems capable of targeted delivery of porphyrin derivatives could be useful since they can lead to their accumulation in the target sites and prevent adverse events at the neighboring tissues [130].

More specificity to eliminate malignant cells via photodamage could also be achieved through controlling the light irradiation for the activation of the photosensitizer [132]. Additionally, after the NCL-mediated internalization of the aptamer-photosensitizer complex and its entry to the nucleus of the NCL-overexpressing malignant cells, the photosensitizer could be released from the complex without breaking the covalent bond to target the telomeric DNA or DNA duplex of oncogene promoters which results in the induction of telomerase inhibition or blockade of oncogene transcription [130].

Moreover, Shen et al. [133] have shown the potential of using a combination of tumor-targeting and ATP-binding aptamers by incorporating them into hybrid micellar NPs to design ATP-activatable photosensitizers for imaging and cancer PDT. They used amine-functionalized hybrid micellar NPs, termed NH2-HyNP, and customized it with AS1411, a BHQ_2_-labeled ATP-binding aptamer (termed BHQ_2_-ATP-apt), and the complementary oligonucleotide c-TA for ATP-apt to make their delivery platform (Apt-HyNP/BHQ_2_) [133].

**Figure 4.**
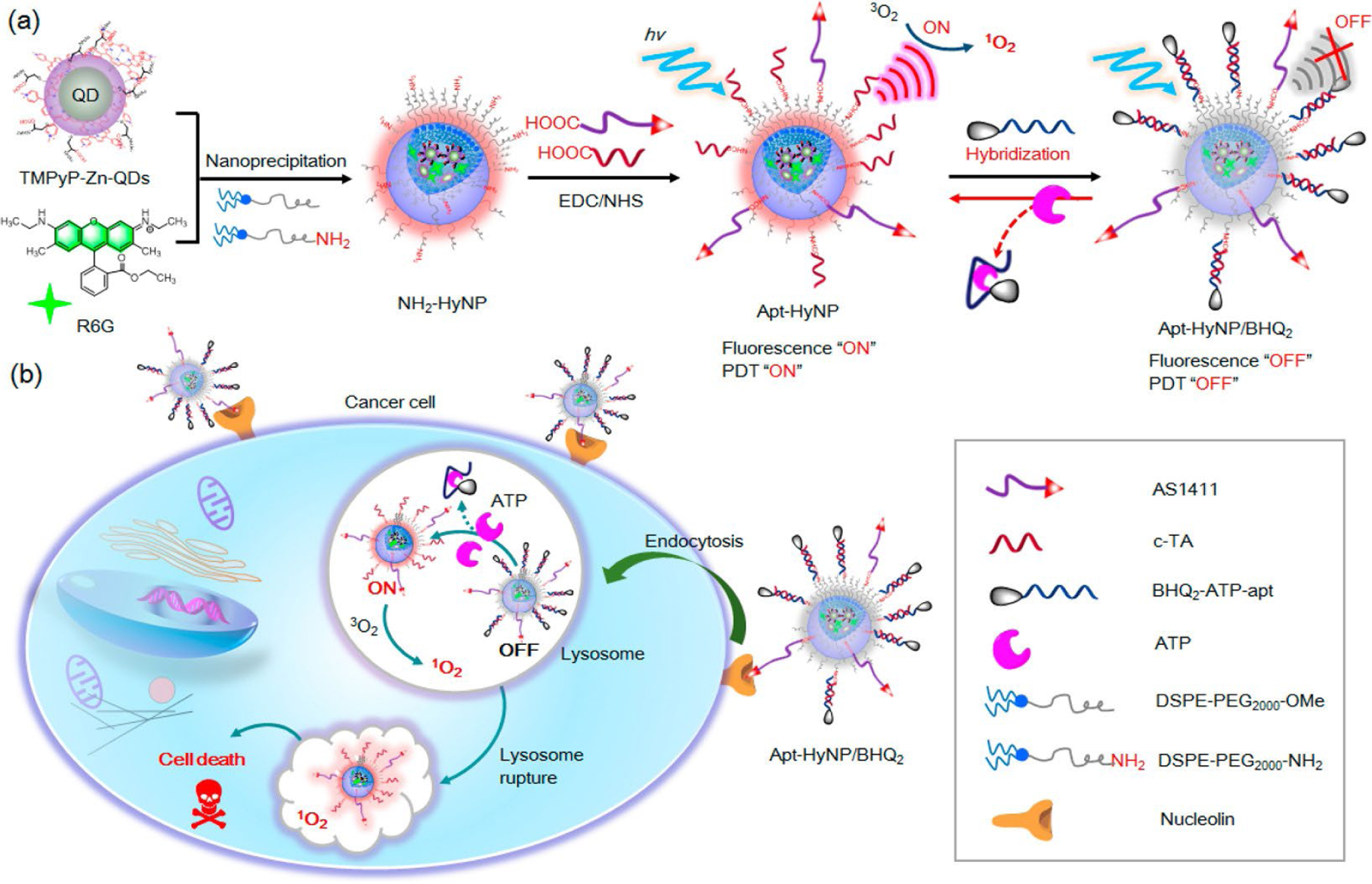
The design of tumor-targeting and ATP-activatable photosensitizer, Apt-HyNP/BHQ2, for fluorescence imaging and cancer PDT. (a) The schematic representation of the procedure for the synthesis of Apt-HyNP/BHQ2; (b) An illustration of the mechanism of action of Apt-HyNP/BHQ2 prior to and after NCL-mediated endocytosis. R6G: rhodamine 6G *Note.* Adapted with permission from “ATP-activatable photosensitizer enables dual fluorescence imaging and targeted photodynamic therapy of tumor” by Shen Y, Tian Q, Sun Y, Xu JJ, Ye D, Chen HY. Anal Chem. 2017;89:13610–7 (https://doi.org/10.1021/acs.analchem.7b04197). Copyright (2017) American Chemical Society.

Due to the quenching effect of BHQ_2_, Apt-HyNP/BHQ_2_ is fluorescence and PDT “off” in the beginning [133]. After entering the malignant tissues, AS1411 interacts with the cell surface NCL which leads to efficient endocytosis and selective accumulation of Apt-HyNP/BHQ_2_ in the lysosomes of the cells [133]. The high concentration of intracellular ATP can specifically bind to the BHQ_2_-ATP-apt, therefore, cause them to be released from Apt-HyNP/BHQ_2_ which leads to turning “on” both fluorescence and PDT with significant recovery of both fluorescence emissions and ^1^O_2_ production capacity [133]. Therefore, the irradiation of tumor cells could trigger significant ^1^O_2_ generation which in turn would lead to rapid lysosome rupture and ultimate tumor cell death (Figure 4). As a result, this approach might serve as a tumor-targeting and ATP-activatable photosensitizer with enhanced tumor selectivity for accurate cancer PDT without noticeable side effects [133].

**Figure 5.**
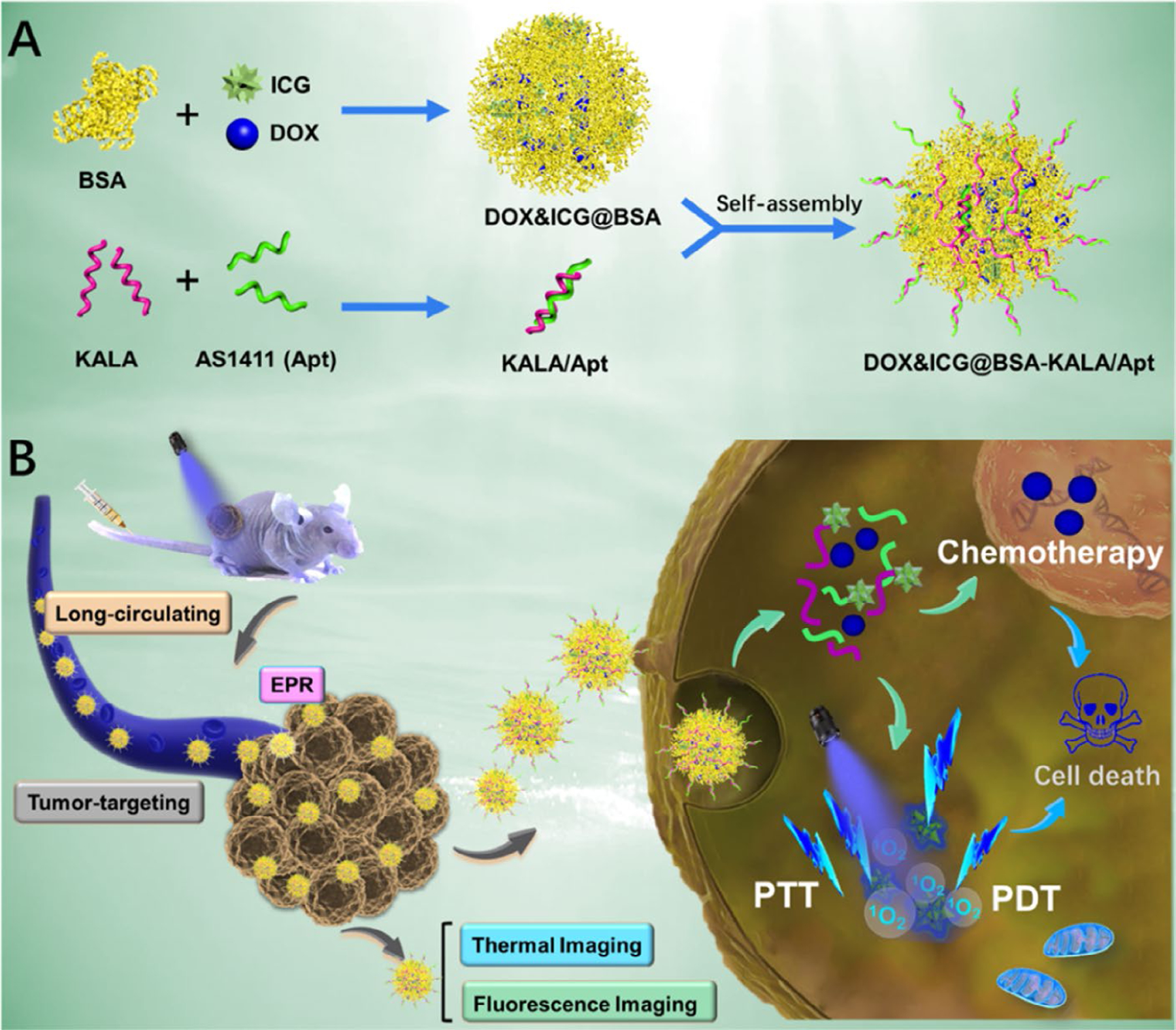
Schematic Illustration of (A) the preparation procedure and (B) the theranostic process of DOX&ICG@BSA-KALA/Apt NPs. PTT: photothermal therapy *Note.* Adapted with permission from “Multifunctional albumin-based delivery system generated by programmed assembly for tumor-targeted multimodal therapy and imaging” by Xu L, Wang SB, Xu C, Han D, Ren XH, Zhang XZ, et al. ACS Appl Mater Interfaces. 2019;11:38385–94 (https://doi.org/10.1021/acsami.9b11263). Copyright (2019) American Chemical Society.

Furthermore, various studies have combined other approaches such as targeted-delivery of drugs and photosensitizers. In this regard, Xu et al. [134] have proposed an elaborate strategy by developing a nano-sized protein-based multimodal theranostic system harboring ideal immunocompatibility and biodegradability to integrate chemotherapy and PDT. In this system, DOX and the phototherapeutic agent indocyanine green (ICG) are utilized as hydrophobic drugs to self-assemble with bovine serum albumin (BSA) molecules to form nano-sized particles [134]. The mentioned particles are subsequently surface-decorated with the AS1411 aptamer and a peptide named KALA which possesses a considerable cell penetration ability [134]. The cellular uptake of the resultant NPs is significantly improved because of their surface-decoration with AS1411 and KALA leading to a more efficient tumor-targeted multimodal therapy [134]. After the internalization of the resultant NPs, which happens upon the interaction between AS1411 and the cell surface NCL and is also facilitated by KALA, the release of the loaded DOX and ICG occurs [134]. On one hand, DOX plays its chemotherapeutic role and leads to cell death [134]. On the other hand, under laser irradiation, the singlet oxygen generation capability of ICG enables it to produce intracellular singlet oxygen molecules which in turn facilitate the cellular apoptotic pathway (Figure 5) [134]. In conclusion, this strategy has shown that effective integration of PDT and chemotherapy can lead to an optimized therapeutic efficacy with minimized side effects [134].

## Conclusion

In this article, we reviewed the studies using the AS1411 aptamer for functionalizing and redirecting NPs loaded with chemotherapeutic agents or therapeutic nucleotides towards various types of NCL-overexpressing cancer cells. We also discussed how these aptamer-functionalized nanosystems can selectively deliver their payload only to tumor cells, therefore, reducing the off-target delivery-associated toxicities of conventional cancer therapy treatment modalities. Furthermore, several studies have shown that chemical modifications performed on AS1411 can result in augmented functionality, increased tumor cell targeting affinity, enhanced S-phase cell cycle arrest capability, and improved DNA replication and cancer cell growth prohibition ability of the aptamer [135, 136]. These outcomes propose that the position and number of modification substituents in AS1411 are important factors for enhancing the diagnostic and therapeutic function of the aptamer [135, 136]. Aptamer-mediated NCL-targeting has also been used in other fields such as imaging probes for tracking tumor cells [137, 138] as well as in quick chip assay for the capturing of circulating tumor cells [139]. Overall, it is safe to say that, as emerging targeting agents, aptamers are quickly being recognized as fighting tools against different types of cancers, which alongside having commercial potential, might pave the troubled way of cancer treatment and diagnosis. Delivering different types of cargoes with the AS1411 aptamer and targeting them to NCL-overexpressing cells have already demonstrated to be a promising strategy and it might mature into a more innovative hope-delivery system for cancer patients in the near future.

## Figures and Tables

**Table 1.** A summary of AS1411-functionalized chemotherapeutic drug delivery nanosystems for targeted cancer therapy

Delivery system	Components	Drug(s)	Animal models or/and cells lines	Investigated cancer type	*In vivo/in vitro*	Reference
NP	PLGA-Lecithin-PEG	PTX	MCF-7, GI-1	Breast cancer and gliosarcoma	*In vitro*	[59]
NP	MF-NR-PLGA	PTX	MCF-7	Breast cancer	*In vitro*	[60]
NP	PEG-PCL	Docetaxel	BALB/c mice & BALB/c nude mice/C6, bEnd.3	Glioma	*In vivo*/*in vitro*	[61]
NP	PLGA-PEG-COOH	PTX	C6 glioma xenograft mice, intracranial C6 glioma rats	Glioma	*In vivo*	[62]
NP	pPEGMA-PCL-pPEGMA	DOX	MCF-7, PANC-1	Epithelial cancer	*In vitro*	[63]
NP	HSA	PTX	MCF-7	Breast cancer	*In vitro*	[64]
NP	PLGA-PEG	Vinorelbine	MDA-MB-231	Breast cancer	*In vitro*	[65]
NP	Cytochrome C capped mesoporous silica NPs	DOX	Athymic BALB/c nude mice/HepG2	Hepatocellular carcinoma	*In vivo*/*in vitro*	[66]
Nanosphere	Colloidal gold nanospheres	-	Fox1^nu^ nude mice/MCF-7 & MDA-MB-231	Breast cancer	*In vivo*/*in vitro*	[67]
Nanostar	Gold nanostar	-	Nude mice, Sprague-Dawley (SD) rats/HT-1080, MDA-MB-231	Fibrosarcoma, breast cancer	*In vivo*/*in vitro*	[68]
Drug-DNA adduct	-	DOX	NOD.Cg-*Prkdc*^*SCID*^ IL2 mice/Huh7	Hepatocellular carcinoma	*In vivo/in vitro*	[69]
Nanotube	SWCNT	DOX	BALB/c nude mice/PC3	Prostate cancer	*In vivo/in vitro*	[70]
Micelles	Pluronic F127, β-CD-PELA	DOX	BALB/c nude mice/MCF-7	Breast cancer	*In vivo/in vitro*	[47]
Micelles	TPGS-b-PBAE	PTX	BALB/c nude mice/SKOV3	Ovarian cancer	*In vivo/in vitro*	[52]
Capsule	Cell membrane capsules	DOX hydrochloride	BALB/c nude mice/QGY-7703	Liver cancer	*In vivo/in vitro*	[71]
Micelles	PEG-PAE	DOX	MCF-7	Breast cancer	*In vitro*	[53]

MF: magnetic fluorescence; NR: Nile red; PCL: polycaprolactone; pPEGMA: poly(polyethylene glycol methacrylate); SWCNT: single walled carbon nanotubes; PBAE: blockpoly-(β-amino ester)

## Abbreviations

ALGDG2: anionic linear globular dendrimer G2
DOX: doxorubicin
EDC: ethyl(dimethylaminopropyl) carbodiimide
EMT: epithelial-mesenchymal transition
EpCAM: epithelial cell adhesion molecule
EPR: enhanced permeability and retention
GSH: glutathione
IAPs: inhibitors of apoptosis
ICG: indocyanine green
MF: magnetic fluorescence
NCL: nucleolin
NHS: *N*-hydroxysuccinimide
NP: nanoparticle
NR: Nile red
NRP1: neuropilin 1
NSCLC: non-small cell lung cancer
PAE: poly(β-amino esters)
PCL: polycaprolactone
PDT: photodynamic therapy
PEG: polyethylene glycol
pPEGMA: poly(polyethylene glycol methacrylate)
PTX: paclitaxel
RB: retinoblastoma
SELEX: Systematic Evolution of Ligands by EXponential Enrichment
siBraf: BRAF siRNA
SLUG: snail family zinc finger 2
SSO: splice switching oligonucleotide
Sur: survivin
Sur_Dz: survivin DNAzyme
TPGS: tocopheryl polyethylene glycol 1000 succinate
β-CD-PELA: beta-cyclodextrin-linked poly(ethylene glycol)-b-polylactide
